# The interplay of ADHD characteristics and executive functioning with the GPA and divergent thinking of engineering students: A conceptual replication and extension

**DOI:** 10.3389/fpsyg.2022.937153

**Published:** 2022-07-27

**Authors:** Christa L. Taylor, Arash Esmaili Zaghi

**Affiliations:** ^1^Psychological Sciences Research Institute, Université catholique de Louvain, Louvain-la-Neuve, Belgium; ^2^Department of Civil and Environmental Engineering, University of Connecticut, Storrs, CT, United States

**Keywords:** attention deficit hyperactivity disorder, executive functioning, divergent thinking, academic performance, GPA

## Abstract

Characteristics of attention-deficit hyperactivity disorder (ADHD) and executive functioning difficulties have been found to correspond with poorer academic outcomes on the one hand and enhanced divergent thinking on the other hand. The current study was conducted to better understand the relationship between ADHD characteristics, executive functioning difficulties, divergent thinking, and academic outcomes by conceptually replicating and expanding on a previous study. Undergraduate engineering students (*N* = 199) at a public university in the northeastern United States completed self-report measures of ADHD characteristics and daily executive functioning, as well as divergent thinking (figural and verbal) and intelligence quotient (IQ) tests. The results of a series of multiple regression models showed that (1) executive functioning difficulties negatively, and non-verbal IQ and figural divergent thinking positively, predicted engineering grade point average (GPA; obtained from the university registrar’s office), (2) GPA and verbal IQ positively predicted figural divergent thinking scores, and (3) verbal IQ positively predicted verbal divergent thinking scores. A series of multiple regression models testing the assertion that controlling for IQ would strengthen the relationship between divergent thinking and ADHD characteristics or executive functioning were not supported but did show associations between select components of characteristics and divergent thinking. Taken together, these results support previous conclusions that students with ADHD characteristics and executive functioning difficulties may struggle academically yet exhibit select enhanced divergent thinking abilities.

## Introduction

Recent meta-analytic evidence suggests that 506.27 million adults worldwide meet the diagnostic criteria for attention-deficit hyperactivity disorder (ADHD; Song et al., 2021). Although there are 2.58% of adults with ADHD worldwide who were diagnosed as children, a further 6.76% of adults who were not diagnosed as children nonetheless experience clinically significant characteristics of ADHD (Song et al., 2021). Such characteristics have been suggested to arise from difficulties with executive functioning (i.e., the management of cognitive functions; Barkley, 1997) and include difficulty concentrating, restlessness, and poor organization and planning (Brown, 2006). These characteristics and executive functioning difficulties correspond with poorer academic outcomes for college students, even in cases where students do not meet the diagnostic criteria for ADHD (Biederman et al., 2006; Petersen et al., 2006; Norwalk et al., 2009; Munro et al., 2017). However, these characteristics have also been found to correspond with enhanced performance on divergent thinking tasks, which assess one’s ability to generate many original responses to a stimulus (Zabelina et al., 2014; Boot et al., 2017; Taylor et al., 2020b). Understanding both the challenges and strengths of students with ADHD characteristics and executive functioning difficulties is necessary to create a more holistic view of learners and provide insights into how we can best support students that may not fit into the standard definition of “normal” cognitive functioning. Therefore, the purpose of this study was to better understand the relationship between ADHD characteristics and executive functioning difficulties, divergent thinking, and academic performance by conceptually replicating and expanding on a recent study (Taylor et al., 2020b) that found that ADHD characteristics negatively predicted major grade point average (GPA) and positively predicted figural divergent thinking in engineering students.

### Attention-deficit hyperactivity disorder and executive functioning

Attention-deficit hyperactivity disorder is characterized by a persistent pattern of inattention and/or hyperactivity–impulsivity that impacts one’s daily life (American Psychiatric Association, 2013; World Health Organization, 2022). A clinical diagnosis of ADHD in adults is based on their experience of a set of characteristics (i.e., symptoms) from the inattentive dimension, characterized by difficulties with sustaining attention (e.g., being easily distracted or not completing tasks), and/or hyperactive–impulsive dimension, characterized by difficulties with inhibiting behaviors (e.g., interrupting others or not being able to remain still). The fifth edition of the Diagnostic and Statistical Manual for Mental Disorders (i.e., DSM-5; American Psychiatric Association, 2013) suggests that at least five characteristics from either or both dimensions must have been present in multiple settings for at least 6 months, be inconsistent with the person’s developmental level, and negatively impact their occupational, social, and/or educational functioning. Additionally, several of the characteristics must have been present before the age of 12. Of note, the DSM-5 diagnostic criteria for ADHD have been criticized as being too lax, artificially inflating diagnoses (Rigler et al., 2016; Fabiano and Haslam, 2020), and ethnocentric, making it an inappropriate assessment tool in some cultures (Kriegler, 2015; Berri and Al-Hroub, 2016; Shehab and Al-Hroub, 2019).

Although a diagnostic interview is necessary for a clinical diagnosis of ADHD, self-report screening measures developed for use in the community and workplace (e.g., Kessler et al., 2005; Barkley, 2011; Ustun et al., 2017) are frequently used in psychological research to understand how ADHD characteristics vary with other constructs. For example, the ADHD Self-Report Screening Scale (ASRS; Kessler et al., 2005; Ustun et al., 2017) was developed by creating self-report items closely following DSM criteria for ADHD, in addition to items assessing executive functioning difficulties frequently displayed by those with ADHD. The Barkley Deficits in Executive Functioning Scale (B-DEFS; Barkley, 2011) explicitly focuses on items developed from the executive functioning theory of ADHD, which suggests that characteristics of ADHD arise from impaired executive functioning (Barkley, 1997; Brown, 2006; Antshel et al., 2014).

Attention-deficit hyperactivity disorder has been found to be strongly associated with difficulties in executive functioning, a group of higher-order cognitive functions that manage cognitive processing, such as inhibition, shifting, and working memory (Nigg et al., 2005; Willcutt et al., 2005; Stavro et al., 2007; Silverstein et al., 2020). Adults with ADHD perform more poorly than others on laboratory tasks measuring specific components of executive functions, such as inhibiting prepotent responses on the Stroop Color–Word Test (Murphy et al., 2001; Boonstra et al., 2005; Nigg et al., 2005; Willcutt et al., 2005; Stavro et al., 2007). Although laboratory tasks may be more objective measures of specific executive functions, self- or other-report assessments of executive functioning have been suggested to be more ecologically valid and to better predict functional outcomes (e.g., occupational, social, and educational outcomes; Barkley and Fischer, 2011; Barkley and Murphy, 2011) for several reasons. First, adults with ADHD who have a high intelligence quotient (IQ) may not demonstrate impairments on laboratory tests of executive functioning (Biederman et al., 2008; Milioni et al., 2017). Second, laboratory tests of executive functions may be contaminated by non-executive functioning cognitive processes or general cognitive ability (Burgess et al., 2006; Biederman et al., 2008). Third, laboratory tests are typically conducted to assess a narrow set of executive functions, which provide little insight into how the daily behavioral manifestations of executive functioning ability relate to functional outcomes.

Indeed, individuals with ADHD score higher on self-report measures of executive functioning difficulties than others and executive functioning difficulties are positively associated with the severity of ADHD characteristics (Biederman et al., 2006, 2007; Barkley and Fischer, 2011; Barkley and Murphy, 2011; Silverstein et al., 2020). For example, Silverstein et al. (2020) found that the severity of ADHD (diagnosed using the DSM-5 semi-structured clinical interview) was strongly and positively associated with self-report ratings of executive functioning difficulties on the Behavior Rating Inventory of Executive Function (Roth et al., 2005). Self-reported executive functioning difficulties are also associated with functional outcomes in those that do not meet the criteria for a diagnosis of ADHD (Brown and Casey, 2016; Vélez-Pastrana et al., 2016; Dehili et al., 2017; Kamradt et al., 2019), suggesting that the characteristics of ADHD and executive functioning difficulties may impact the daily life of many more individuals.

### Attention-deficit hyperactivity disorder, executive functioning, and academic outcomes

Students with ADHD have poorer academic outcomes in general than students without ADHD (Frazier et al., 2007; Dupaul et al., 2009; Dipeolu, 2011). College students with ADHD have been found to have lower GPAs and are more likely to be on academic probation (Heiligenstein et al., 1999; Frazier et al., 2007). Further, academic outcomes have been found to be poorer for students with ADHD who also exhibit poorer executive functioning, assessed using a series of cognitive tasks, compared to students with ADHD who scored higher on the tasks (Biederman et al., 2006). However, ADHD characteristics and executive functioning difficulties are associated with poorer academic outcomes even for those without ADHD (Biederman et al., 2006).

Research shows that ADHD characteristics and executive functioning difficulties are also associated with poorer academic outcomes in the general (i.e., non-clinical) student population (Petersen et al., 2006; Norwalk et al., 2009; Knouse et al., 2014; Munro et al., 2017). For example, Knouse et al. (2014) found that lower cumulative GPA was associated with self-reported executive functioning difficulties in undergraduate students at two different universities. Academic adjustment (e.g., the ability to cope with the challenges of college life) and study habits and skills (e.g., completing assigned class readings) have also been negatively predicted by self-reported characteristics of ADHD in a general student population (Norwalk et al., 2009). Indeed, poorer executive functioning predicts a number of characteristics that may hamper academic success, such as procrastination (Rabin et al., 2011; Rinaldi et al., 2019) and lack of motivation (Knouse et al., 2014).

### Attention-deficit hyperactivity disorder, executive functioning, and divergent thinking

Divergent thinking refers to the generation of many original and diverse responses to a stimulus (Guilford, 1956, 1967). Although convergent thinking, narrowing down ideas and solutions by discarding inappropriate or less desirable ones, also plays a role, divergent thinking ability is critical for solving novel problems (Guilford, 1987). This ability is supported by four processes: (1) fluency, quickly generating ideas, (2) flexibility, generating diverse ideas, (3) originality, generating unique ideas, and (4) elaboration, developing ideas. Divergent thinking, reflecting the capacity for idea generation, is a critical component of creativity (Reiter-Palmon et al., 2019) and has been shown to relate to a wide range of positive outcomes, such as personal resiliency and higher grades (Metzl, 2009; Gajda et al., 2017).

How divergent thinking relates to ADHD characteristics and executive functioning difficulties in adults remains uncertain. Whereas some studies found evidence for enhanced divergent thinking ability in college students diagnosed with ADHD compared to students not diagnosed with ADHD (White and Shah, 2006, 2011, 2016), other studies and meta-analyses found no association (Barkley et al., 1996; Paek et al., 2016). However, studies more consistently found divergent thinking ability to be positively associated with self-reported ADHD characteristics and executive functioning difficulties in non-clinical samples (Zabelina et al., 2014; Boot et al., 2017; Hoogman et al., 2020; Taylor et al., 2020a,b). Boot et al. (2017) found that college students’ self-reported experience of characteristics of ADHD positively predicted originality on a problem construction task, which asked participants to redefine an everyday problem at different levels of usefulness and originality. Taylor et al. (2020a) found that components of executive functioning difficulties related to ADHD (i.e., activation, affect, attention, effort, and memory; T. E. Brown, 1996b) predicted different facets of divergent thinking in unique ways. For example, controlling for all other components, affect (i.e., problems regulating one’s mood and motivation) predicted fluency, whereas activation (i.e., problems with volition, organization, and prioritizing) predicted originality, on the Torrance Tests of Creative Thinking (TTCT)-Figural. Thus, although the relationship between clinically diagnosed ADHD and divergent thinking remains uncertain, studies more consistently show that higher (compared to lower) self-reported ADHD characteristics and executive functioning difficulties are associated with better performance on divergent thinking tasks in adults (see Hoogman et al., 2020).

### The present study

The current literature suggests that ADHD characteristics and executive functioning difficulties correspond with poorer academic outcomes on the one hand (Frazier et al., 2007; Dupaul et al., 2009; Dipeolu, 2011) and enhanced divergent thinking on the other hand (Zabelina et al., 2014; Boot et al., 2017; Taylor et al., 2020b). The relationship between ADHD and executive functioning difficulties, academic outcomes, and divergent thinking has only previously been examined together in one study. Taylor et al. (2020b) demonstrated that ADHD characteristics can predict figural divergent thinking scores in engineering students, yet negatively predict engineering GPA. These findings suggest that college students with ADHD and related characteristics may struggle academically, while their potential strengths remain overlooked in traditional academic programs.

However, the Taylor et al. (2020b) study was limited in several important ways. First, the sample size was relatively small, making the meaning of non-significant results ambiguous. Second, participants were aware of the purpose of the study and data collection was not blinded, which may have heightened the risk of bias on the ADHD scales. Third, although the association between divergent thinking and other variables has been shown to be influenced by the modality of assessment (i.e., figural vs. verbal), only figural divergent thinking was assessed. Fourth, the measure used to assess ADHD characteristics has not been widely used in research or validated in a general student population. Fifth, intelligence (IQ), which has been suggested to affect the relationship between ADHD and divergent thinking ability, was not measured.

Understanding this relationship may be particularly important for engineering because the structure of post-secondary engineering education differs from that of other fields in ways that may make it more demanding for those with executive functioning difficulties (Veenstra et al., 2009). These difficulties may contribute to poorer academic performance (i.e., lower GPA), which influences later opportunities (e.g., admission to graduate school; Stemler, 2012; Chari and Potvin, 2019). However, undergraduate engineering GPA is a poor predictor of later success in the field of engineering (Samson et al., 1984; Bretz, 1989), for which divergent thinking and related abilities are key (National Academy of Engineering, 2005; Cropley, 2015; Brunhaver et al., 2017; Passow and Passow, 2017).

Thus, to better understand the relationship between ADHD characteristics and executive functioning difficulties, divergent thinking, and academic performance, the current study presents a conceptual replication of Taylor et al. (2020b) that addresses these limitations by: (1) including a larger sample of participants; (2) ensuring better validity of the measure of ADHD characteristics (i.e., obscuring the purpose of the study and ADHD characteristics); (3) assessing both figural and verbal divergent thinking; (4) using more widely used measures of ADHD characteristics and executive functioning difficulties; and (5) measuring verbal (i.e., crystallized) and non-verbal (i.e., fluid) intelligence. Based on the results of Taylor et al. (2020b), we expect that:

*Hypothesis 1:* Executive functioning difficulties will negatively predict engineering GPA.

*Hypothesis 2:* Executive functioning difficulties will positively predict divergent thinking, whereas engineering GPA will negatively predict divergent thinking.

In addition, we expand on this research by examining the role of IQ in the relationship between modalities of divergent thinking (figural and verbal) and the components of ADHD assessed by one measure focused explicitly on executive functioning difficulties associated with ADHD (B-DEFS) and one measure that aligns with DSM and WHO diagnostic criteria (ASRS). If these scales are measuring the same underlying phenomenon, results for both scales should be broadly similar. Therefore, we expect that:

*Hypothesis 3*: The relationship between ADHD characteristics on the B-DEFS and divergent thinking will be stronger when controlling for IQ.

*Hypothesis 4*: The relationship between ADHD characteristics on the ASRS and divergent thinking will be stronger when controlling for IQ.

## Materials and methods

This study was approved by the institutional review board at the participating university (protocol #H17-196).

### Participants

Undergraduate engineering students at a public university in the northeastern United States were recruited to participate in the study with flyers and emails sent to their student account. Participants were compensated with a $35 gift card (prorated at $8.75 per session) after completing or withdrawing from the study. Of 220 students who participated, 20 withdrew from the study and one was discovered to not be eligible for participation (i.e., was under the age of 18), resulting in a final sample of 199 participants (56.3% men and 43.7% women). Participants’ age ranged from 18 to 33 years (*M* = 19.89, *SD* = 1.86), and their grade level classification was distributed as follows: 32 sophomores, 49 juniors, and 118 seniors.

### Materials

#### Barkley deficits in executive functioning scale

The B-DEFS (Barkley, 2011) assesses EF impairments associated with ADHD across five dimensions: time management, self-motivation, self-restraint, self-organization/problem-solving, and self-regulation of emotion. The scale was developed and validated using a nationally representative sample of adults in the United States (Barkley, 2011) and has been shown to validly assess daily EF across a large non-clinical sample of college students across five universities in the United States (Kamradt et al., 2019). Participants are asked to indicate, on a four-point scale from 1 (never or rarely) to 4 (very often), how often they have experienced each of 89 observable behaviors reflecting EF impairments within the previous 6 months, for example, having trouble doing things in a proper order or doing something without first considering the consequences. A total scale score is obtained by summing the scores for all items, whereas subscale scores are obtained by summing the items associated with each of the five dimensions. In the current study, internal consistency was strong for the B-DEFS total scale (Cronbach’s α = 0.96) and for each of the subscales: time management (α = 0.93), self-motivation (α = 0.87), self-restraint (α = 0.85), self-organization/problem-solving (α = 0.91), and self-regulation of emotion (α = 0.83).

#### Adult attention-deficit hyperactivity disorder self-report scale symptom checklist

The ASRS (Kessler et al., 2005) assesses the characteristics (i.e., symptoms) of ADHD, based on the criteria for adult ADHD in the Diagnostic and Statistical Manual of Mental Disorders, fourth edition (American Psychiatric Association, 1994). The scale was developed by the World Health Organization and has been shown to correspond strongly with a clinician-administered assessment of ADHD characteristics (Adler et al., 2006). Participants were asked to indicate, on a five-point scale from 0 (never) to 4 (very often), how often they have experienced each of 18 characteristics of ADHD within the previous 6 months. Nine items on the scale reflect the inattentive dimension of ADHD, such as forgetting appointments and obligations, and nine items reflect the hyperactive/impulsive dimension, such as interrupting when others are speaking. Scores for each dimension are obtained by summing the items corresponding to each. In the current study, internal consistency was acceptable for the inattentive (α = 0.77) and hyperactive/impulsive (α = 0.71) subscales.

#### Torrance tests of creative thinking

The TTCT (Torrance, 2008) assesses divergent thinking using either a verbal or figural response format. The TTCT-Verbal test contains five activities that ask participants to provide a written response, such as providing as many creative uses for a cardboard box as possible, within a given time limit (ranging from 5 to 10 min). Responses on the TTCT-Verbal were rated on three dimensions: fluency (total number of responses), originality (infrequency of responses), and flexibility (variability in categories of responses). The TTCT-Figural test contains three activities that ask participants to provide a drawn response, such as providing as many creative pictures from a series of parallel lines as possible, with a time limit of 10 min for each task. Responses on the TTCT-Figural are rated on five dimensions: fluency (total number of responses), originality (infrequency of responses), elaboration (detail of responses), resistance to premature closure (lack of constraint of responses), and titles (abstractness of the title provided for responses). In the current study, responses on both modalities of the TTCT (both Form A), scored by three trained and certified raters at Scholastic Testing Services (STS), demonstrated strong inter-rater reliability, according to Cronbach’s α: TTCT-Verbal (fluency = 1.00, originality = 1.00, flexibility = 0.98) and TTCT-Figural (fluency = 0.99, originality = 0.99, elaboration = 0.98, resistance to premature closure = 1.00, and titles = 0.99). Thus, raters’ scores were averaged to create total dimension scores for each participant. In addition, total TTCT scores were created for each form by averaging the scores for each corresponding dimension (after *z*-transformation based on the sample, to account for differing rating scales).

#### Kaufman brief intelligence test, second edition

The Kaufman Brief Intelligence Test, Second Edition (KBIT-2) (Kaufman and Kaufman, 2004) assesses verbal and non-verbal IQ across three brief subtests: verbal knowledge, matrices, and riddles. The number of items that a participant completes on each subtest is determined by a start point, which is determined by the participant’s age and a stop rule based on incorrect responding. The verbal knowledge subtest contains 60 items and asks participants to indicate which of six pictures presented on an easel corresponds with a word read by an administrator. The matrices subtest contains 46 items and asks participants to indicate which of five pictures corresponds with a concept conveyed by a stimulus or which of six pictures completes a presented matrix. The riddles subtest contains 39 items and asks participants to verbally respond with the correct word to a riddle read by an administrator. Three scores may be computed according to the testing manual: a general composite IQ score, a verbal IQ score, based on scores for the verbal knowledge and riddles subtests, and a non-verbal IQ score, based on the score for the matrices subtest.

### Procedure

Participants completed the study in a private office across four 30-to-60-min sessions on different days. In the first session, participants completed informed consent, followed by the TTCT-Figural test. In the second session, participants used a tablet to complete the B-DEFS and ASRS (along with items from several other scales for use in a different study) on Qualtrics, with all scale items presented in a random order. In the third session, participants completed an engineering design activity (for use in a different study) followed by the KBIT-2, administered by a trained researcher. In the fourth session, participants completed the TTCT-Verbal test. Following completion of the study, cumulative engineering GPA was provided by the university’s Office of the Registrar.

### Statistical analysis

Data were analyzed using IBM SPSS Statistics, version 27. Data for one student who did not complete the IQ test were excluded from analyses involving IQ scores. There were no other missing data. Descriptive statistics (mean, median, standard deviation, minimum and maximum values, skew, and kurtosis) were obtained for all variables included in the analyses. Bivariate correlations were analyzed using Pearson correlations (α = 0.05) with pair-wise deletion excluding data for univariate outliers (>±3.5 SD from the mean). Hypotheses were tested using a series of multiple regression models (α = 0.05), with data for outliers (std. residual >±3) excluded from the corresponding models.

## Results

Descriptive statistics for all variables are shown in Table 1. Bivariate correlations among the variables (Table 2) were calculated excluding data for two outliers on the B-DEFS total scale and one outlier each on verbal IQ, non-verbal IQ, B-DEFS time, B-DEFS organization, B-DEFS self-restraint, B-DEFS motivation, B-DEFS emotional regulation, and TTCT-Figural originality. However, distributions for several variables deviated substantially from normal, according to the standard scores of skewness and kurtosis (score divided by standard error >3.3; Tabachnick and Fidell, 2007) and the Shapiro–Wilk test (*p* > 0.01) even after removing data for univariate outliers. Therefore, bivariate correlations among the variables were also examined using complete data with Spearman’s rank correlations. There were no appreciable differences in the values between the contrasting correlations, and conclusions based on the results remained the same.

**TABLE 1:** Descriptive statistics for all variables included in the analyses.

Variable	Mean	Median	SD	Min.	Max.	Skew	Kurtosis
Engineering GPA	3.26	3.38	0.56	1.10	4.00	−1.03	0.91
IQ Verbal*^a^*	107.58	107	12.94	66	145	0.00	0.58
IQ Non-verbal*^a^*	108.14	111	13.65	70	132	−0.40	−0.15
B-DEFS Total	157.34	153	34.79	98	296	0.92	1.22
B-DEFS Time	38.27	35	11.12	21	78	1.13	1.14
B-DEFS Organization	44.64	43	11.63	24	88	0.48	0.12
B-DEFS Self-restraint	31.73	30	7.84	19	60	0.98	0.98
B-DEFS Motivation	20.26	18	6.02	12	44	1.03	0.71
B-DEFS Emotion Regulation	22.44	22	5.94	13	43	0.55	0.03
ASRS Inattentive	25.20	25	5.51	12	43	0.62	0.66
ASRS Hyperactive/impulsive	26.19	26	5.65	13	44	0.34	0.32
TTCT-Figural Total	0.00	−0.02	0.72	−1.76	2.25	0.18	0.00
TTCT-Figural Fluency	18.99	19	6.37	7.00	40.00	0.52	0.23
TTCT-Figural Originality	14.10	13	5.40	4.00	38.00	0.92	1.45
TTCT-Figural Elaboration	9.68	9.33	2.56	3.00	18.00	0.24	0.31
TTCT-Figural Titles	11.79	11	4.64	0.00	22.00	0.11	−0.11
TTCT-Figural Resist. Closure	13.47	14	3.92	4.00	20.00	−0.32	−0.99
TTCT-Verbal Total	0.00	−0.03	0.97	−2.25	2.92	0.30	0.05
TTCT-Verbal Fluency	89.60	86.33	28.82	24.00	175.33	0.46	0.08
TTCT-Verbal Originality	67.55	64.67	25.07	21.33	143.00	0.57	0.00
TTCT-Verbal Flexibility	49.36	49.33	11.14	19.67	80.33	−0.10	−0.03

^a^N = 189; all other N = 199.

**TABLE 2:** Bivariate correlations for all variables included in the analyses.

Variable	1	2	3	4	5	6	7	8	9	10	11	12	13	14	15	16	17	18	19	20	21
1- Engineering GPA	−																				
**IQ**																					
2- Verbal	0.04	−																			
3- Non-verbal	**0.14**	**0.36**	−																		
**B-DEFS**																					
4- Total	**−0.18**	0.02	0.08	−																	
5- Time	**−0.24**	**0.15**	**0.14**	**0.85**	−																
6- Organization	−0.06	**−0.17**	0.06	**0.84**	**0.59**	−															
7- Self-restraint	−0.11	−0.03	0.07	**0.78**	**0.60**	**0.55**	−														
8- Motivation	**−0.31**	0.02	−0.01	**0.83**	**0.78**	**0.60**	**0.55**	−													
9- Emotion Regulation	−0.05	−0.02	0.04	**0.59**	**0.32**	**0.47**	**0.49**	**0.28**	−												
**ASRS**																					
10- Inattentive	−0.12	0.04	0.07	**0.73**	**0.69**	**0.61**	**0.61**	**0.57**	**0.39**	−											
11- Hyper/impulsive	0.05	0.06	0.10	**0.45**	**0.31**	**0.35**	**0.52**	**0.26**	**0.39**	**0.62**	−										
**TTCT-Figural**																					
12- Total	0.13	**0.14**	−0.06	0.02	0.06	−0.07	0.05	−0.03	0.05	0.06	0.06	−									
13- Fluency	**0.16**	−0.07	−0.10	−0.06	−0.03	−0.07	0.03	−0.04	0.01	0.02	0.00	**0.76**	−								
14- Originality	0.09	0.13	−0.02	0.05	0.11	−0.02	0.09	−0.01	−0.02	0.05	0.01	**0.77**	**0.68**	−							
15- Elaboration	0.08	**0.33**	0.06	−0.06	0.08	**−0.13**	−0.03	−0.04	0.00	0.05	0.08	**0.60**	**0.28**	**0.32**	−						
16- Titles	0.05	**0.16**	0.00	0.00	0.05	−0.02	0.03	0.00	0.10	0.05	0.08	**0.69**	**0.26**	**0.26**	**0.39**	−					
17- Resist Closure	0.12	−0.01	−0.11	0.02	0.04	0.02	0.10	−0.01	0.09	0.06	0.04	**0.79**	**0.58**	**0.54**	0.18	**0.57**	−				
**TTCT-Verbal**																					
18- Total	0.06	**0.26**	0.01	−0.12	−0.10	**−0.18**	−0.11	**−0.14**	0.01	0.01	0.12	**0.44**	**0.32**	**0.39**	**0.51**	**0.22**	0.13	−			
19- Fluency	0.0	**0.23**	−0.01	−0.12	−0.11	**−0.18**	−0.10	**−0.15**	0.01	0.00	0.12	**0.43**	**0.33**	**0.38**	**0.48**	**0.21**	0.13	**0.98**	−		
20- Originality	0.06	**0.22**	0.01	−0.12	−0.11	**−0.18**	−0.11	**−0.14**	0.00	0.01	0.13	**0.41**	**0.31**	**0.37**	**0.47**	**0.20**	0.12	**0.97**	**0.97**	−	
21- Flexibility	0.11	**0.30**	0.04	−0.11	−0.08	**−0.17**	−0.11	−0.13	0.01	0.01	0.11	**0.43**	**0.31**	**0.37**	**0.53**	**0.23**	**0.14**	**0.94**	**0.88**	**0.86**	−

All coefficients in bold are significant at p < 0.05.

### Conceptual replication

Several multiple regression models were tested to conceptually replicate those of Taylor et al. (2020b). Results and coefficients for all models are shown in Table 3. Assumptions were met for all models, after removing three outlying cases from the first model. The first model, wherein all predictors were entered into the model simultaneously, was statistically significant, predicting 10% of the variability in engineering GPA. Engineering GPA was significantly, positively predicted by TTCT-Figural scores and non-verbal IQ and negatively predicted by B-DEFS scores. For each identical model predicting the two modalities of the TTCT, predictors were entered simultaneously with either TTCT-Figural or TTCT-Verbal scores as the outcome. The model predicting TTCT-Figural scores was statistically significant with verbal IQ and engineering GPA significantly, positively predicting TTCT-Figural scores. The model predicting TTCT-Verbal scores was also statistically significant with verbal IQ as the only significant, positive predictor.

**TABLE 3:** Predictors of engineering GPA, TTCT-Figural, and TTCT-Verbal scores.

	Model			
Variable	*B (SE)*	β	*t*	*P*
**Engineering GPA**				
B-DEFS	−0.004 (0.001)	−0.27	−3.82	<0.001
IQ-Non-verbal	0.007 (0.003)	0.20	2.68	0.01
IQ-Verbal	−0.002 (0.003)	−0.04	−0.55	0.58
TTCT-Figural	0.141 (0.055)	0.20	2.58	0.01
TTCT-Verbal	−0.015 (0.042)	0.03	−0.35	0.73
	*F*(5, 189) = 5.19, *p* < 0.001, *R*^2^ = 0.12, adj. *R*^2^ = 0.10			
**TTCT-Figural**				
B-DEFS	0.001 (0.002)	0.07	0.98	0.33
Engineering GPA	0.213 (0.094)	0.17	2.26	0.03
IQ-Non-verbal	−0.008 (0.004)	−0.15	−2.02	0.05
IQ-Verbal	0.011 (0.004)	0.19	2.57	0.01
	*F*(4, 193) = 3.05, *p* = 0.02, *R*^2^ = 0.06, adj. *R*^2^ = 0.04			
**TTCT-Verbal**				
B-DEFS	−0.003 (0.002)	−0.12	−1.74	0.08
Engineering GPA	0.052 (0.124)	0.03	0.42	0.67
IQ-Non-verbal	−0.006 (0.005)	−0.08	−1.11	0.27
IQ-Verbal	0.021 (0.006)	0.28	3.87	<0.001
	*F*(4, 193) = 4.85, *p* = 0.001, *R*^2^ = 0.09, adj. *R*^2^ = 0.07			

### The relationship between components of divergent thinking and attention-deficit hyperactivity disorder characteristics

A series of hierarchical regression models were used to examine how components of the B-DEFS (i.e., time management, self-motivation, self-restraint, self-organization/problem-solving, and self-regulation of emotion) or ASRS (i.e., inattentive and hyperactive/impulsive) predict the components of figural and verbal divergent thinking, controlling for IQ. Given that, theoretically, controlling for IQ should increase the strength of the relationship between scores on the B-DEFS (and ASRS) scales and components of divergent thinking, scale variables were entered in step one and verbal IQ and non-verbal IQ were entered in step two in all models. Assumptions were met for all models, after removing outlying cases from several of the models as indicated below.

#### Barkley deficits in executive functioning scale

Results and coefficients for all models predicting TTCT-Verbal scores are shown in Table 4. The models for TTCT-Verbal fluency, originality, and flexibility (after removing one outlying case: std. residual = −3.05) were not statistically significant for B-DEFS components alone (step 1) but were significant after adding verbal and non-verbal IQ (step 2). The final models explained between 5 and 11% of variance in TTCT-Verbal component scores, with verbal IQ as the only significant, positive predictor in all cases.

**TABLE 4:** Hierarchical regression models of B-DEFS and IQ as predictors of TTCT-Verbal components.

	Model 1		Model 2	
Variable	*B (SE)*	β	*B (SE)*	β
**Fluency**				
Time management	0.289 (0.327)	0.11	−0.020 (0.347)	−0.01
Motivation	−0.565 (0.610)	−0.12	−0.418 (0.611)	−0.09
Self-restraint	0.078 (0.368)	0.02	0.142 (0.363)	0.04
Organization	−0.636 (0.249)	−0.26*	−0.395 (0.259)	−0.16
Emotion Regulation	0.465 (0.419)	0.10	0.399 (0.413)	0.08
IQ-Non-verbal			−0.192 (0.159)	−0.09
IQ-Verbal			0.525 (0.181)	0.24**
*F*	2.20		2.84	
*R* ^2^	0.05		0.10	
adj. *R*^2^	0.03		0.06	
Δ*F*			4.26*	
Δ*R*^2^			0.04	
**Originality**				
Time management	0.294 (0.284)	0.13	0.040 (0.303)	0.02
Motivation	−0.474 (0.530)	−0.11	−0.339 (0.534)	−0.08
Self-restraint	0.013 (0.320)	0.00	0.060 (0.317)	0.02
Organization	−0.565 (0.217)	−0.26*	−0.376 (0.226)	−0.17
Emotion Regulation	0.394 (0.365)	0.09	0.345 (0.361)	0.08
IQ-Non-verbal			−0.122 (0.139)	−0.07
IQ-Verbal			0.411 (0.158)	0.21*
*F*	2.23		2.60*	
*R* ^2^	0.06		0.09	
adj. *R*^2^	0.03		0.05	
Δ*F*			3.40*	
Δ*R*^2^			0.03	
**Flexibility**				
Time management	0.151 (0.124)	0.15	−0.043 (0.129)	−0.04
Motivation	−0.208 (0.232)	−0.12	−0.090 (0.227)	−0.05
Self-restraint	0.019 (0.141)	0.01	0.048 (0.135)	0.03
Organization	−0.253 (0.095)	−0.27**	−0.118 (0.097)	−0.13
Emotion Regulation	0.178 (0.161)	0.10	0.142 (0.154)	0.08
IQ-Non-verbal			−0.040 (0.059)	−0.05
IQ-Verbal			0.286 (0.067)	0.34***
*F*	2.11		4.28	
*R* ^2^	0.05		0.14	
adj. *R*^2^	0.03		0.11	
Δ*F*			9.25***	
Δ*R*^2^			0.08	

*p < 0.05, **p < 0.01, ***p < 0.001.

Results and coefficients for all models predicting TTCT-Figural scores are shown in Table 5. The models for TTCT-Figural fluency (for which two outlying cases were removed: std. residual = 3.05 and 3.31), titles, and resistance to closure were not statistically significant for B-DEFS components (step 1) or after adding verbal and non-verbal IQ (step 2). The models for TTCT-Figural originality (for which two outlying cases were removed: std. residual = 3.03 and 4.38) and elaboration were statistically significant for B-DEFS components (step 1), as well as after adding verbal and non-verbal IQ (step 2). However, whereas B-DEFS time was a positive predictor, and B-DEFS motivation a negative predictor, in the final model for TTCT-Figural originality scores, verbal IQ was the only significant predictor in the final model for TTCT-Elaboration scores.

**TABLE 5:** Hierarchical regression models of B-DEFS and IQ as predictors of TTCT-Figural components.

	Model 1		Model 2	
Variable	*B (SE)*	β	*B (SE)*	β
**Fluency**				
Time management	−0.038 (0.070)	−0.07	0.000 (0.076)	0.00
Motivation	0.017 (0.131)	0.02	−0.026 (0.133)	−0.03
Self-restraint	−0.030 (0.081)	−0.04	−0.026 (0.081)	−0.03
Organization	−0.012 (0.054)	−0.02	−0.026 (0.057)	−0.05
Emotion Regulation	0.071 (0.090)	0.07	0.070 (0.090)	0.07
IQ-Non-verbal			−0.042 (0.035)	−0.09
IQ-Verbal			−0.028 (0.039)	−0.06
*F*	0.30		0.61	
*R* ^2^	0.01		0.02	
adj. *R*^2^	−0.02		−0.01	
Δ*F*			1.39	
Δ*R*^2^			0.25	
**Originality**				
Time management	0.173 (0.057)	0.38**	0.149 (0.061)	0.33*
Motivation	−0.248 (0.106)	−0.30*	−0.241 (0.108)	−0.29*
Self-restraint	0.090 (0.064)	0.14	0.096 (0.064)	0.15
Organization	−0.037 (0.044)	−0.09	−0.017 (0.046)	−0.04
Emotion Regulation	−0.081 (0.073)	−0.10	−0.087 (0.073)	−0.10
IQ-Non-verbal			−0.023 (0.028)	−0.06
IQ-Verbal			0.045 (0.032)	0.12
*F*	2.70*		2.23*	
*R* ^2^	0.07		0.08	
adj. *R*^2^	0.04		0.04	
Δ*F*			1.06	
Δ*R*^2^			0.01	
**Elaboration**				
Time management	0.066 (0.029)	0.29*	0.027 (0.030)	0.12
Motivation	−0.008 (0.054)	−0.02	0.017 (0.053)	0.04
Self-restraint	−0.014 (0.032)	−0.04	−0.009 (0.032)	−0.03
Organization	−0.069 (0.022)	−0.31**	−0.042 (0.023)	−0.19
Emotion Regulation	0.024 (0.037)	0.06	0.017 (0.036)	0.04
IQ-Non-verbal			−0.009 (0.014)	−0.05
IQ-Verbal			0.058 (0.016)	0.30***
*F*	2.71*		4.08***	
*R* ^2^	0.07		0.13	
adj. *R*^2^	0.04		0.10	
Δ*F*			7.09**	
Δ*R*^2^			0.07	
**Resistance to Closure**				
Time management	−0.008 (0.045)	−0.02	0.000 (0.049)	−0.00
Motivation	−0.030 (0.085)	−0.05	−0.050 (0.086)	−0.08
Self-restraint	0.044 (0.051)	0.09	0.050 (0.051)	0.10
Organization	−0.004 (0.035)	−0.01	0.001 (0.036)	0.00
Emotion Regulation	0.063 (0.058)	0.10	0.059 (0.058)	0.09
IQ-Non-verbal			−0.038 (0.022)	−0.13
IQ-Verbal			0.012 (0.25)	0.04
*F*	0.64		0.89	
*R* ^2^	0.02		0.03	
adj. *R*^2^	−0.01		−0.00	
Δ*F*			1.49	
Δ*R*^2^			0.02	
**Titles**				
Time management	0.029 (0.054)	0.070	−0.009 (0.057)	−0.02
Motivation	0.031 (0.100)	0.04	0.051 (0.101)	0.07
Self-restraint	−0.032 (0.060)	−0.05	−0.024 (0.060)	−0.04
Organization	−0.054 (0.041)	−0.14	−0.025 (0.043)	−0.06
Emotion Regulation	0.122 (0.069)	0.16	0.114 (0.068)	0.15
IQ-Non-verbal			−0.020 (0.026)	−0.06
IQ-Verbal			0.063 (0.030)	0.18*
*F*	0.85		1.24	
*R* ^2^	0.02		0.04	
adj. *R*^2^	0.00		0.01	
Δ*F*			2.20	
Δ*R*^2^			0.02	

*p < 0.05, **p < 0.01, ***p < 0.001.

#### Attention-deficit hyperactivity disorder self-report scale

Results and coefficients for all models predicting TTCT-Verbal scores are shown in Table 6. The models for TTCT-Verbal fluency, originality, and flexibility (after removing two outlying cases: std. residual = 3.30 and −3.30) were not statistically significant for ASRS components alone (step 1) but were significant after adding verbal and non-verbal IQ (step 2). The final models explained between 6 and 11% of variance in TTCT-Verbal component scores, with verbal IQ and the hyperactive/impulsive dimension of the ASRS significantly, positively predicting TTCT-Verbal components in all cases.

**TABLE 6:** Hierarchical regression models of ASRS and IQ as predictors of TTCT-Verbal components.

	Model 1		Model 2	
Variable	*B (SE)*	β	*B (SE)*	β
**Fluency**				
Inattentive	−0.642 (0.473)	−0.12	−0.64	−0.12
Hyperactive/impulsive	0.990 (0.461)	0.19*	0.971 (0.450)	0.19*
IQ-Non-verbal			−0.243 (0.156)	−0.12
IQ-Verbal			0.582 (0.164)	0.26***
*F*	2.31		4.38**	
*R* ^2^	0.02		0.08	
adj. *R*^2^	0.01		0.06	
Δ*F*			6.33**	
Δ*R*^2^			0.06	
**Originality**				
Inattentive	−0.557 (0.411)	−0.12	−0.559 (0.402)	−0.12
Hyperactive/impulsive	0.920 (0.400)	0.21*	0.898 (0.393)	0.20*
IQ-Non-verbal			−0.172 (0.136)	−0.09
IQ-Verbal			0.476 (0.143)	0.25**
*F*	2.65		4.15**	
*R* ^2^	0.03		0.08	
adj. *R*^2^	0.02		0.06	
Δ*F*			5.53**	
Δ*R*^2^			0.05	
**Flexibility**				
Inattentive	−0.209 (0.180)	−0.11	−0.214 (0.170)	−0.11
Hyperactive/impulsive	0.359 (0.176)	0.19*	0.338 (0.167)	0.18*
IQ-Non-verbal			−0.057 (0.058)	−0.07
IQ-Verbal			0.298 (0.061)	0.35***
*F*	2.10		7.28***	
*R* ^2^	0.02		0.13	
adj. *R*^2^	0.01		0.11	
Δ*F*			12.23***	
Δ*R*^2^			0.11	

*p < 0.05, **p < 0.01, ***p < 0.001.

Results and coefficients for all models predicting TTCT-Figural scores are shown in Table 7. The models for TTCT-Figural fluency (for which two outlying cases were removed: std. residual = 3.24 and 3.36), originality (for which one outlying case was removed: std. residual = 4.32), titles, and resistance to closure were not statistically significant for ASRS components (step 1) or after adding verbal and non-verbal IQ (step 2). The model for TTCT-Figural elaboration was not statistically significant for ASRS components alone (step 1) but was significant after adding verbal and non-verbal IQ (step 2). The final model explained 10% of variance, with verbal IQ as the only significant, positive predictor of TTCT-Figural elaboration.

**TABLE 7:** Hierarchical regression models of ASRS and IQ as predictors of TTCT-Figural components.

	Model 1		Model 2	
Variable	*B (SE)*	β	*B (SE)*	β
**Fluency**				
Inattentive	−0.014 (0.102)	−0.01	−0.015 (0.101)	−0.01
Hyperactive/impulsive	0.001 (0.098)	0.00	0.015 (0.098)	0.01
IQ-Non-verbal			−0.045 (0.034)	−0.10
IQ-Verbal			−0.023 (0.036)	−0.05
***F***	0.01		0.78	
***R* ^2^**	0.00		0.02	
**adj. *R*^2^**	−0.01		−0.00	
**Δ*F***			0.01	
**Δ*R*^2^**			1.55	
**Originality**				
Inattentive	0.089 (0.084)	0.10	0.089 (0.083)	0.10
Hyperactive/impulsive	−0.047 (0.082)	−0.05	−0.053 (0.081)	−0.06
IQ-Non-verbal			−0.012 (0.029)	−0.03
IQ-Verbal			0.066 (0.029)	0.17*
***F***	0.56		1.56	
***R* ^2^**	0.01		0.03	
**adj. *R*^2^**	−0.01		0.01	
**Δ*F***			2.56	
**Δ*R*^2^**			0.03	
**Elaboration**				
Inattentive	−0.001 (0.042)	−0.00	−0.001 (0.040)	−0.00
Hyperactive/impulsive	0.036 (0.041)	0.08	0.030 (0.039)	0.07
IQ-Non-verbal			−0.013 (0.014)	−0.07
IQ-Verbal			0.069 (0.014)	0.35**
***F***	0.62		6.40**	
***R* ^2^**	0.01		0.12	
**adj. *R*^2^**	−0.00		0.10	
**Δ*F***			12.12**	
**Δ*R*^2^**			0.11	
**Resistance to Closure**				
Inattentive	0.043 (0.065)	0.06	0.044 (0.065)	0.06
Hyperactive/impulsive	−0.001 (0.063)	−0.00	0.006 (0.063)	0.01
IQ-Non-verbal			−0.036 (0.022)	−0.12
IQ-Verbal			−0.009 (0.023)	0.03
***F***	0.35		0.83	
***R* ^2^**	0.00		0.02	
**adj. *R*^2^**	−0.01		−0.00	
**Δ*F***			1.32	
**Δ*R*^2^**			0.01	
**Titles**				
Inattentive	0.005 (0.077)	0.01	0.005 (0.076)	0.01
Hyperactive/impulsive	0.064 (0.075)	0.08	0.061 (0.074)	0.08
IQ-Non-verbal			−0.025 (0.026)	−0.07
IQ-Verbal			0.064 (0.027)	0.18*
***F***	0.66		1.72	
***R* ^2^**	0.01		0.03	
**adj. *R*^2^**	−0.00		0.01	
**Δ*F***			2.78	
**Δ*R*^2^**			0.03	

*p < 0.05, **p < 0.001.

## Discussion

The purpose of this study was 2-fold. First, we sought to better understand how ADHD characteristics and executive functioning difficulties, divergent thinking, and engineering GPA relate to one another. Taylor et al. (2020b) provided insight into this matter by showing that ADHD characteristics negatively predicted engineering GPA, yet positively predicted figural divergent thinking. However, the conclusions of this study were limited by several methodological issues (e.g., small sample size and assessing figural divergent thinking only). Therefore, we conducted a conceptual replication of Taylor et al. (2020b) that addresses these limitations. Second, we sought to better understand the role of IQ in the relationship between ADHD characteristics or executive functioning difficulties and divergent thinking. Although it has been frequently suggested that the relationship between ADHD characteristics and divergent thinking may be strengthened by accounting for IQ (e.g., Boot et al., 2017; Taylor et al., 2020a), IQ has rarely been assessed in studies examining this relationship (though see Taylor and Zaghi, 2021). Therefore, we also examined whether relationships between components of the B-DEFS or dimensions of the ASRS and components of the verbal and figural TTCT were strengthened after controlling for IQ.

The results of our conceptual replication of Taylor et al. (2020b) were partially consistent with hypotheses derived from the results of that study. Consistent with Hypothesis 1, the model predicting GPA from executive functioning difficulties, divergent thinking, and IQ was significant, with scores on the B-DEFS negatively predicting GPA. Additionally, in contrast to the results of Taylor et al. (2020b), scores on the TTCT-Figural positively predicted GPA along with non-verbal IQ. Hypothesis 2, which suggested that executive functioning difficulties would positively—and GPA would negatively—predict divergent thinking, was not supported for models predicting figural or verbal TTCT scores. Rather, GPA and verbal IQ positively predicted TTCT-Figural scores, and verbal IQ alone positively predicted TTCT-Verbal scores. Therefore, this study supports some of the conclusions of Taylor et al. (2020b) using different methods, such as the negative relationship between executive functioning difficulties and engineering GPA. However, understanding the causes of discrepant results, such as the surprising finding in our study that engineering GPA positively predicted TTCT-Figural scores, will require further research.

Our hypotheses that controlling for IQ would strengthen the relationship between divergent thinking and executive functioning difficulties (Hypothesis 3) or ADHD characteristics measured by the ASRS (Hypothesis 4) were not supported. Not surprisingly, given the strength of the correlations between fluency, originality, and flexibility on the TTCT-Verbal, results for models predicting these scores using the same predictors were nearly identical. Although components of the B-DEFS alone did not significantly predict scores on any component of the TTCT-Verbal, models were significant after adding IQ, with verbal IQ emerging as the only significant positive predictor in all three cases. Although the ASRS dimensions (i.e., inattentive and hyperactive/impulsive) did not significantly predict any TTCT-Verbal scores alone, models were significant after adding IQ, with both verbal IQ and the hyperactive/impulsive dimension of the ASRS positively predicting all three scores. Taken together, these results demonstrate two things: First, the relationship between components of divergent thinking on the TTCT-Verbal and inattentive characteristics related to ADHD is broadly similar using either the B-DEFS or the ASRS. Thus, the B-DEFS and the inattentive dimension of the ASRS may indeed assess similar behaviors, ostensibly stemming from executive functioning difficulties related to attention. Second, hyperactive/impulsive (though not inattentive) characteristics of ADHD predict TTCT scores, even after accounting for IQ. This aligns with Boot et al. (2017) findings that positive associations between performance on verbal divergent thinking tasks and self-reported ADHD characteristics were primarily driven by hyperactive/impulsive characteristics.

Results for models predicting component scores on the TTCT-Figural were also broadly similar whether ADHD characteristics were measured using the B-DEFS or the ASRS in most cases. ADHD characteristics did not predict fluency, titles, or resistance to closure, either alone or after including IQ, and elaboration was significantly predicted by verbal IQ only. However, results for the models predicting originality scores on the TTCT-Figural differed when including the B-DEFS dimensions or the ASRS dimensions as predictors. Although ASRS scores did not predict originality either alone or after controlling for IQ, B-DEFS scores did significantly predict originality scores both alone and after controlling for IQ. Although the model explained only a small amount of variance in originality scores (4%), difficulty with time management positively predicted originality and difficulty with motivation negatively predicted originality. This is consistent with Taylor et al.’s (2020a) finding that scores on the activation subscale of the Brown ADD Scales (Brown, 1996a), which indicates difficulties related to time management (e.g., difficulties with procrastinating, organizing and prioritizing tasks, and estimating time), positively predicted originality on the TTCT-Figural. Thus, although controlling for IQ did not strengthen the relationship between ADHD characteristics and components of divergent thinking, several of our models were significant in ways consistent with existing evidence.

Our results, along with existing evidence, point to several key issues in understanding how engineering GPA, ADHD characteristics or executive functioning difficulties, and divergent thinking relate to one another. First, engineering GPA can be predicted by executive functioning difficulties, IQ, and divergent thinking. Controlling for other variables, behaviors reflecting strong executive functioning, non-verbal IQ, and figural divergent thinking were positively associated with GPA. Second, the relationship between engineering GPA and figural divergent thinking may be stronger than previously thought. Although TTCT-Figural scores were not a significant predictor of engineering GPA in Taylor et al. (2020b) study, the effect was in the same direction and only slightly smaller than that found in the current study (i.e., 0.16 and 0.20, respectively), suggesting that the null result found in that study could have indeed been due to inadequate power stemming from the small sample size (*N* = 50). Third, verbal and figural divergent thinking may relate to different dimensions of ADHD characteristics and executive functioning difficulties in distinct ways. Our results align with those of Boot et al. (2017) in finding that verbal divergent thinking abilities may relate to hyperactive/impulsive characteristics and with those of Taylor et al. (2020a) in finding that figural divergent thinking originality relates to select inattentive characteristics. This is consistent with evidence that different modalities of divergent thinking draw on different abilities and skills (Clapham, 2011; Barbot, 2018).

### Limitations and future research directions

Although the current study fills important gaps in our understanding of how ADHD characteristics and executive functioning difficulties, divergent thinking, and academic performance relate to one another, there are several limitations that can be addressed in future research. First, we are unable to pinpoint the causes of discrepancies in the results of our conceptual replication and those of Taylor et al. (2020b). There are several potential reasons for why, in contrast to Taylor et al. (2020b), executive functioning difficulties were not a significant predictor of figural divergent thinking in the current study. Perhaps, the most salient is our use of a different measure to capture executive functioning difficulties. To determine whether the discrepancy in these results is due to the use of different self-reported executive functioning scales, future studies could administer the Brown ADD scales along with the B-DEFS.

Second, we did not include a clinical diagnostic assessment for ADHD, which limits the generalizability of our results to the general population. There is evidence that, because the primary characteristics of ADHD are distributed in the population, ADHD is best conceptualized as the extreme end of a dimension (as opposed to categorical; e.g., Lubke et al., 2009; Heidbreder, 2015; Brown and Casey, 2016). However, to determine whether our results are relevant for those diagnosed with ADHD, future studies should recruit students with an existing ADHD diagnosis and/or include a clinical diagnostic assessment.

Third, the amount of variance explained by some of the models predicting components of divergent thinking is rather small, despite being statistically significant. For example, although the model was statistically significant, B-DEFS and IQ scores accounted for only 4% of variability in originality scores on the TTCT-Figural. Thus, caution should be exercised when drawing conclusions based on the results of these models. However, future research could also examine whether other factors (e.g., personality or social support) influence how ADHD characteristics and executive functioning difficulties predict divergent thinking.

### Conclusion

The current study was conducted to better understand the relationship between ADHD characteristics and executive functioning difficulties, divergent thinking, and academic performance. Results align with previous research showing that executive functioning difficulties are associated with poorer engineering GPA. However, results contradict previous research by finding that figural divergent thinking ability is associated with better engineering GPA. Additionally, hyperactive/impulsive ADHD characteristics were associated with greater verbal divergent thinking, whereas select executive functioning difficulties (primarily reflecting inattentive characteristics) were associated with greater figural divergent thinking originality. Although the relevance of these results for students in other academic fields requires further study, engineering students who struggle academically due to executive functioning difficulties associated with ADHD may benefit from focusing on their strengths related to figural divergent thinking.

## Data availability statement

The raw data supporting the conclusion of this article will be made available by the authors, without undue reservation.

## Ethics statement

The studies involving human participants were reviewed and approved by Institutional Review Board at the University of Connecticut. The patients/participants provided their written informed consent to participate in this study.

## Author contributions

CT and AZ contributed to the conception and design of the study. AZ managed the data collection. CT performed the statistical analysis and wrote the first draft of the manuscript. Both authors contributed to manuscript revision and read and approved the submitted version.
